# Complex Intra-Operonic Dynamics Mediated by a Small RNA in *Streptomyces coelicolor*

**DOI:** 10.1371/journal.pone.0085856

**Published:** 2014-01-20

**Authors:** Hindra, Matthew J. Moody, Stephanie E. Jones, Marie A. Elliot

**Affiliations:** Department of Biology and Institute for Infectious Disease Research, McMaster University, Hamilton, Ontario, Canada; University of Strathclyde, United Kingdom

## Abstract

*Streptomyces* are predominantly soil-dwelling bacteria that are best known for their multicellular life cycle and their prodigious metabolic capabilities. They are also renowned for their regulatory capacity and flexibility, with each species encoding >60 sigma factors, a multitude of transcription factors, and an increasing number of small regulatory RNAs. Here, we describe our characterization of a conserved small RNA (sRNA), scr4677. In the model species *Streptomyces coelicolor,* this sRNA is located in the intergenic region separating *SCO4677* (an anti-sigma factor-encoding gene) and *SCO4676* (a putative regulatory protein-encoding gene), close to the *SCO4676* translation start site in an antisense orientation. There appears to be considerable genetic interplay between these different gene products, with wild type expression of scr4677 requiring function of the anti-sigma factor SCO4677, and scr4677 in turn influencing the abundance of *SCO4676*-associated transcripts. The scr4677-mediated effects were independent of RNase III (a double stranded RNA-specific nuclease), with RNase III having an unexpectedly positive influence on the level of *SCO4676*-associated transcripts. We have shown that both SCO4676 and SCO4677 affect the production of the blue-pigmented antibiotic actinorhodin under specific growth conditions, and that this activity appears to be independent of scr4677.

## Introduction

*Streptomyces* bacteria are best known for their complex developmental life cycle and their vast secondary metabolic capabilities, which include producing the majority of naturally synthesized antibiotics. The *Streptomyces* life cycle involves transitioning between several differentiated states (reviewed in [1], [2]), and initiates with the germination of a single spore. Germ tube elongation and subsequent hyphal tip extension and branching lead to the formation of a filamentous cellular network known as the vegetative or substrate mycelium. Reproductive aerial hyphae are raised from the vegetative mycelium, with these aerial structures ultimately being converted into chains of dormant exospores. Aerial hyphae formation is coupled, both genetically and temporally, with the onset of secondary metabolism and antibiotic production [3]. In the model organism *Streptomyces coelicolor*, the different growth and metabolic stages are readily distinguishable. During growth on agar media, aerial hyphae formation results in a fuzzy white colony appearance, while sporulation culminates with the production of a grey polyketide pigment that turns colonies grey [4]. Equivalent visual cues accompany antibiotic production, courtesy of the fact that *S. coelicolor* produces both blue and red pigmented antibiotics (actinorhodin and undecylprodigiosin, respectively).

The streptomycetes are predominantly found in the soil and have large genomes, ranging in size from 7–12 Mbp [5], [6], [7]. This presumably provides them with the genetic flexibility to adapt and respond to diverse environmental stresses. Consistent with this proposal is the observation that bacterial species with large genomes (>6 Mbp) typically encode a greater proportion of transcription regulators than those with smaller genomes [8]. Notably, *S. coelicolor* devotes more than 12% of its protein-coding genes to regulation [5], and the number of annotated regulatory non-coding RNAs continues to increase [9], [10], [11], [12], [13].

Like their protein counterparts, non-coding RNAs can have positive or negative regulatory impacts on their cellular targets. In bacteria, small RNAs (sRNAs), which typically range in size from 40–500 nucleotides, are the best studied of the non-coding RNAs. Most sRNAs exert their regulatory effects by either modulating the activity of a target protein, or more commonly, by base-pairing with target mRNAs and affecting their stability or translatability (reviewed in [14]). mRNA-targeting sRNAs can be divided into two categories based on their genomic context: *trans*-encoded regulatory RNAs are expressed at sites distinct from those of their target genes and often share only partial sequence complementarity with their target mRNAs, while *cis*-encoded (antisense) regulatory RNAs are expressed where they act and share complete complementarity with their mRNA targets.

Base pairing between antisense regulatory RNAs and their cognate sense mRNAs frequently leads to the formation of structures that can be recognized by the double strand-specific ribonuclease RNase III (*e.g.* [15], [16], [17]). In *S. coelicolor*, RNase III is a global regulator of antibiotic biosynthesis [18], [19], and it affects the abundance of anywhere between 79 and 200 transcripts, including those of at least two regulatory RNAs [20], [21]. Although it is well-established that RNase III acts as a pleiotropic regulator in *S. coelicolor*, regulatory connections between RNase III and *cis-*encoded regulatory RNAs have not been explored in any depth.

Increasing numbers of chromosomally-encoded antisense RNAs are being identified as a result of genome-wide studies in many bacteria [12], [22], [23]. In the streptomycetes, antisense RNAs have now been reported by a number of research groups (*e.g.* [10], [11], [12], [13]). One non-coding RNA having *cis-*regulatory potential is scr4677, a small RNA transcribed immediately upstream of *SCO4676,* in the intergenic region between *SCO4676* and *SCO4677*. We found there to be intriguing genetic connections between the different products expressed from this locus, with wild type expression of scr4677 requiring the activity of SCO4677, scr4677 affecting the levels of *SCO4676*-associated transcripts, and RNase III unexpectedly having a positive effect on *SCO4676* and *SCO4677* levels while having no effect on scr4677. We found that modulating the levels of scr4677 had no obvious phenotypic consequence, deleting *SCO4676* led to enhanced actinorhodin production under defined growth conditions, and loss of *SCO4677* influenced both sporulation and antibiotic production.

## Materials and Methods

### Bacterial Strains and Growth Conditions

*Streptomyces* and *Escherichia coli* strains used in this study are summarized in **Table 1**. *S. coelicolor* strains were grown at 30°C on R2YE (rich, glucose-containing), minimal medium (MM) supplemented with mannitol or glucose, MYM (maltose, yeast extract, malt extract), SMMS (supplemented minimal medium solid), SFM or SFG (soy flour with mannitol or glucose) agar or in liquid R5 (rich, glucose-containing), YEME-TSB (yeast extract, malt extract – tryptone soya broth), or NMMP (minimal) media, as described previously [24], [25]. *Streptomyces avermitilis* was also grown on MYM agar medium, while *Streptomyces venezuelae* was grown in liquid MYM medium. *E. coli* strains were grown at 37°C in Luria-Bertani (LB) or SOB medium [26], with the exception of *E. coli* BW25113 containing pIJ790 (**Table 1**), which was grown at 30°C.

**Table 1:** Bacterial strains and plasmids used in this study.

*Streptomyces* strains	Genotype, description or use	Reference		
*S. coelicolor* M145	SCP1^−^ SCP2^−^	[24]		
*S. coelicolor* E320	M145 Δ*SCO4676::aac(3)IV*	This work		
*S. coelicolor* E321	M145 Δ*SCO4676*	This work		
*S. coelicolor* E322	M145 Δ*SCO4677::aac(3)IV*	This work		
*S. coelicolor* Δrnc	M145 *rnc::aac(3)IV*	[17]		
*S. avermitilis* MA-4680	Wild type	[6]		
*S. venezuelae* ATCC 10712	Wild type			
***E. coli*** ** strains**				
DH5a	Plasmid construction and general subcloning	Invitrogen		
ET12567/pUZ8002	Generation of methylation-free plasmid DNA and conjugation oforiT-containing plasmids	[45] [46]		
BW25113	Construction of cosmid-based knockouts	[29]		
**Plasmids/Cosmids**				
pIJ790	Temperature sensitive plasmid carrying l-RED genes	[29]		
pIJ2925	General cloning vector	[47]		
pWHM3	High copy number *E. coli/Streptomyces* plasmid	[48]		
StD31	Cosmid for *SCO4676* and *SCO4677* knockouts and PCR amplification	[49]		
pUWLFLP	Plasmid carrying the Flp recombinase-encoding gene	[50]		
pIJ82	Complementation of mutant strains	Gift from H. Kieser		
pMC148	pIJ2925+ *scr4677*	This work		
pMC149	pWHM3+ *scr4677*	This work		
pMC145	pIJ2925+ *SCO4676*	This work		
pMC146	pIJ82+ *SCO4676*	This work		
pRT801	Integrative cloning vector	[51]		
pMC150	pRT801+ *SCO4676-3×FLAG*	This work		

### RNA Isolation and Transcript Analysis

RNA was isolated and northern blot analysis was conducted as described previously [10], [13]. Probes used for detection of scr4677 by northern blotting included 4677-1 and 4677-LNA (locked nucleic acid probe) (Table S1). Detection of the scr4677 homologues in *S. avermitilis* and *S. venezuelae* was achieved using oligonucleotide probes svr3556 and sav3140, respectively (Table S1). S1 nuclease mapping of the *SCO4676* transcription start site was performed as outlined previously [27] with the probe generated by PCR amplification using one radiolabelled oligonucleotide (4676 HOT) and one unlabeled primer (46S177) (Table S1), together with cosmid StD31 (Table 1) as template. Semi-quantitative RT-PCR was conducted as described by Hindra *et al.* [28], using the primers listed in Table S1. The number of amplification cycles was optimized for each primer combination to ensure that the reaction products were being generated during the linear phase of the amplification process (cycle numbers ranged from 15 for 16S rDNA, to 32 for the cDNA for the *SCO4677-SCO4676* read-through transcript). Negative control reactions involved adding RNA (without prior reverse transcription) as template to ensure that any amplification observed was not due to DNA contamination of any reaction component. 16S rRNA- or *rpoB*-specific cDNAs were PCR amplified as controls for RNA levels and RNA integrity. PCR products were separated by electrophoresis on 2% agarose gels and visualized by staining with ethidium bromide. All analyses were conducted in triplicate (at a minimum), using samples from at least two independent RNA time courses.

### Construction of sRNA Overexpression Constructs

The scr4677 overexpression strain was constructed by cloning scr4677 into the high copy E. coli-Streptomyces plasmid pWHM3 (Table 1). scr4677 was PCR amplified using primers 4677–3E and 4676 HOT (Table S1) with cosmid StD31 as template DNA, to give a product that was expected to encompass all associated regulatory elements (extending 159 nt upstream and 188 nt downstream of *scr4677*). The resulting product was first cloned into the *Sma*I site of pIJ2925 to give pMC148, and construct integrity was confirmed by sequencing. *scr4677*, together with its flanking sequences, was then excised as a *Bgl*II fragment and cloned into the *Bam*HI site of pWHM3, to give pMC149. This construct, alongside empty pWHM3, was then passaged through the non-methylating *E. coli* strain ET12567 (**Table 1**) before being introduced into *S. coelicolor* M145 using protoplast transformation [24].

### Deletion Strain Construction and Mutant Complementation

*SCO4676* and *SCO4677* deletion strains were constructed using ReDirect technology [29]. Both genes were individually replaced with an apramycin resistance cassette (amplified using primer pairs KO4676-1/KO4676-2 and KO4677-1/KO4677-2; **Table S1**), first within cosmid StD31, and then in the *S. coelicolor* M145 chromosome through double-crossover homologous recombination. Mutations were confirmed using *Bam*HI and *Xho*I restriction enzyme digestions (cosmid) and by PCR (cosmid and chromosome) using diagnostic primer combinations (upstream and downstream of *SCO4676* or *SCO4677*, where wild type and mutant product sizes differed; and upstream and within the deleted coding sequence, where a product would only be detected if a wild type copy of the gene remained; see **Table S1** for primer details). For *SCO4676*, where gene replacement with an apramycin resistance cassette might exert polar effects on the downstream *SCO4675* gene, the resistance cassette, which was flanked by FRT (Flippase recognition target) sites, was excised from the genome. A plasmid encoding the Flp recombinase enzyme (pUWLFLP) (**Table 1**) was conjugated into the *SCO4676* mutant strain. Resulting exconjugants were screened for loss of apramycin resistance and were subsequently confirmed using PCR (KO 4676-1IN and 4675-2IN; **Table S1**).

Wild type *SCO4676*, together with 261 bp of upstream and 211 bp of downstream sequence, was PCR amplified using phosphorylated oligonucleotides 4677-3E and 4675-2IN (**Table S1**). The amplified fragment was introduced into pIJ2925 digested with *Sma*I. The resulting plasmid clone (pMC145; **Table 1**) was sequenced to confirm *SCO4676* integrity before it was excised using *Bgl*II and introduced into *Bam*HI-digested pIJ82 (**Table 1**). The resulting construct (pMC146; **Table 1**) was introduced into *E. coli* ET12567/pUZ8002, and conjugated into the *SCO4676* deletion mutant, followed by selection for plasmid integration into the chromosome.

### Construction of FLAG-tagged SCO4676 and Detection by Immunoblotting

To determine whether SCO4676 protein levels were impacted by scr4677, we created a C-terminally 3×FLAG-tagged fusion protein (creating an N-terminal fusion was complicated by the existence of *scr4677-*associated regulatory sequences that extended up to ∼150 nt into the *SCO4676* coding sequence). Using pMC145 (pIJ2925 carrying the *SCO4676* complementing sequence; **Table 1**), overlap extension PCR was used to introduce an *Xho*I site at the C-terminal end of the coding sequence (between the sequences encoding the fourth and fifth final residues of the protein). Following digestion with *Xho*I, the linearized plasmid was ligated with a 3×FLAG-cassette with *Xho*I-compatible overhangs. FLAG-tag orientation was initially confirmed by PCR using primers USFLAGXho and 4675-2IN (**Table S1**), and the sequence integrity of the entire construct was verified by sequencing. The *SCO4676-3*×*FLAG* fusion was excised using *Bgl*II, and was introduced into the integrating vector pRT801 digested with *Bam*HI, to give pMC150 (**Table 1**). As described for the complementation construct above, the plasmid was passaged through the methylation deficient *E. coli* ET12567/pUZ8002, conjugated into the unmarked *SCO4676* deletion mutant (E321; **Table 1**), and selected for using apramycin. In parallel, untagged *SCO4676* was cloned into an integrating plasmid vector and introduced into *S. coelicolor* E321 to serve as a negative control.

Mycelium from plate-grown cultures was harvested after 20, 64 or 72 h, and equivalent amounts of biomass were resuspended in lysis buffer (10 mM Tris-HCl pH 7.5, 1 mM EDTA, 1 mM DTT, 10% [v/v] glycerol, and 150 mM NaCl), together with lysozyme (750 µg/mL) and protease inhibitor (Roche), as per the manufacturer’s instructions. The suspensions were sonicated on ice, centrifuged, and the soluble proteins retained. Equal volumes (20 µL) of protein extract and loading dye were mixed, heated at 95°C for 5–15 min before the proteins were separated using SDS-PAGE. Alternatively, soluble proteins were precipitated prior to electrophoresis, using a trichloroacetic acid-acetone mixture (1∶9), before being resuspended in Tris buffer (pH 7.5) such that proteins were concentrated four fold. Size-fractionated proteins were transferred to a PVDF membrane using a semi-dry transfer apparatus (Bio-Rad), and were probed with anti-FLAG antibodies (1/1500 dilution: Cell Signaling Technology), and anti-rabbit secondary antibodies (1/3000 dilution; Cell Signaling Technology) as per Duong et al. [52].

### Phenotypic Comparisons of Wild Type, Mutant and Overexpression Strains

For phenotypic analyses, approximately 10^6^ spores were streaked out on a variety of solid media (outlined above). Pigmented antibiotic production and morphological development were compared among strains over a week-long time course. Antibiotic production was visually assessed during plate growth, while morphological development was assessed both visually and using light microscopy. All experiments were conducted a minimum of three times, using at least two independent spore stocks of all strains.

Spores of different *S. coelicolor* strains were also subjected to various stress conditions. This included exposure to heat (60°C, as per [30]) for up to 15 min, 1% (v/v) SDS, 50 mM EDTA, 30 mg/mL lysozyme, 16 mg/mL vancomycin, 0.1 M dipyridyl, 0.05 M diamide, and 0.1 and 0.25% (v/v) hydrogen peroxide. Briefly, soft nutrient agar (0.4 g nutrient broth and 1.15 g nutrient agar per 100 mL medium) was inoculated with approximately 10^6^ spores. This mixture was overlaid onto nutrient agar plates. Paper disks saturated with 20 µL of each compound were placed onto the solidified soft agar, and the plates were then incubated at 30°C for 1 and 2 days. The diameter of any growth inhibitory zones was measured on both days.

To compare levels of actinorhodin production in liquid-grown cultures, 10 mL YEME-TSB was inoculated with ∼10^7^ spores/mL and these cultures were grown overnight at 30°C in a shaking incubator. Five hundred microliters of overnight culture was then used to inoculate 40 mL of liquid minimal medium (NMMP), and these cultures were grown for a further 72 h. All comparisons were conducted using at least three independently grown cultures. Every 24 hours, duplicate 0.5 mL aliquots were removed, and an equal volume of 2 M KOH was added. Samples were then centrifuged to separate cells (which were dried and weighed) and culture supernatant, which was subjected to absorbance measurement at 633 nm [31].

### Cell Free Extract Preparation and Electrophoretic Mobility Shift Assays (EMSAs)

Cell free extracts were prepared from *S*. *coelicolor* grown on cellophane discs overlaying 250 or 350 mL R2YE agar medium in 1.9 or 3 L glass dishes, respectively. Between 12–20 g of biomass (per harvest) was mixed in lysis buffer (as above) such that 0.9 mL lysis buffer was added per 100 mg biomass. Protease inhibitor (Roche) was then added to the suspension as per the manufacturer’s instructions, followed by lysozyme at a concentration of 2.5 mg/mL. The resulting mixture was incubated on ice for 45 min before being homogenized using a glass tissue grinder, and passaged through a French press (Thermo Scientific) three times. The soluble fraction was separated by centrifugation at 16,000×g at 4°C for 20 min.

Probes used for EMSAs comprised complementary oligonucleotides (**Table S1**) that were first annealed by incubating equimolar amounts of each oligonucleotide in annealing buffer (10 mM Tris-HCl pH 7.5, 1 mM EDTA, and 60 mM NaCl), heating to 95°C for 10 min and then slow-cooling to room temperature. Thirty picomoles of the annealed oligonucleotides were then 5′ end-labelled using [γ-^32^P]ATP.

Each 20 µL-binding reaction consisted of 10 mM Tris-HCl pH 7.5, 1 mM DTT, 1 µg poly dI/dC, 10% (v/v) glycerol, 0.5 to 25 nM [γ-^32^P]-labelled DNA fragment, and varying amounts of fresh cell-free extract. Competition assays were conducted by adding 1 µM of either unlabeled probe DNA (specific competitor), or 1 µM of unlabeled non-specific competitor DNA (from within *SCO3287*; **Table S1**; [28]). After being incubated at 30°C for 25–30 min, samples were separated on a non- denaturing 8% polyacrylamide gel. Electrophoresis was performed in 1×TBE buffer at 100 V for 50–60 min. Gels were then transferred to filter paper, wrapped using plastic wrap, and exposed to Kodak Biomax XAR films for 1 h at room temperature.

## Results

### Comparative and Bioinformatics Analysis of scr4677 and its Surrounding Genetic Region

**Figure 1:**
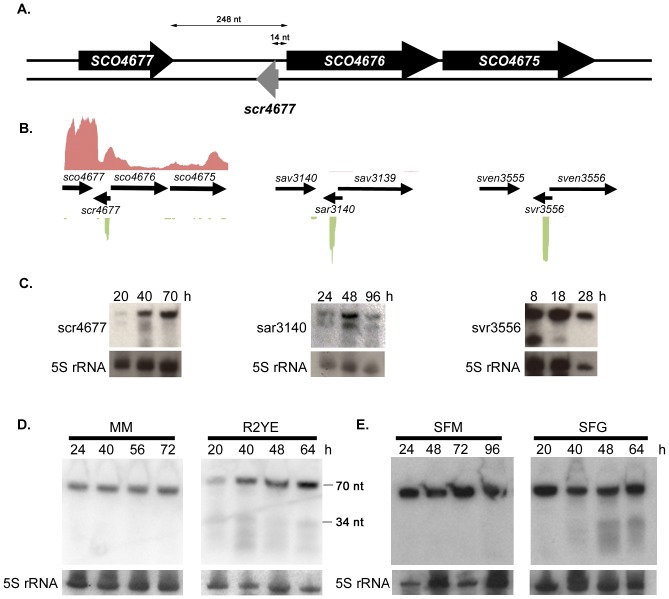
Organization, conservation and expression of scr4677. A) Schematic illustration (not to scale) of the genetic region surrounding *scr4677. scr4677* is located 14 nt upstream of *SCO4676*, in the 248 nt intergenic region between *SCO4677* and *SCO4676*, encoded on the strand opposite these two protein-coding genes. B) and C) *scr4677* is found upstream of *SCO4676* orthologues in many *Streptomyces* species, including *S. coelicolor* (left, where *SCO4676* is co-transcribed with both *SCO4677* and *SCO4675*), *S. avermitilis* (middle) and *S. venezuelae* (right). In B), the green colour denotes gene expression from the negative (antisense) strand, while red indicates expression from the positive (sense) stand. In C) expression of scr4677 (left) and its orthologues sar3140 (middle) and svr3556 (right) was investigated using RNA harvested throughout the developmental cycle (first timepoint: vegetative growth; second timepoint: aerial hyphae/fragmentation; third timepoint: sporulation) using northern blot analysis. D) Northern blot analysis of scr4677 expression in *S. coelicolor* following growth on minimal medium (MM) or rich (R2YE) medium for the times indicated. E) Northern blot analysis of scr4677 expression in *S. coelicolor* following growth on soy flour medium supplemented either with mannitol (SFM; left) or glucose (SFG; right), for the times indicated. For each northern blot, 5S rRNA served as the control for RNA loading and RNA integrity.

In a previous study investigating small RNAs in S. coelicolor, we identified a sRNA termed scr4677 (where ‘scr’ stands for S. coelicolor RNA) [10]. Comparative analyses using select Streptomyces genome sequences available through StrepDB, the Broad Institute and NCBI websites suggested that scr4677 was widely conserved in the streptomycetes, being found in ∼60% of genomes surveyed (Table 2). In S. coelicolor, scr4677 was flanked by SCO4677 and SCO4676, the latter of which appeared to be the first gene of a two-gene operon (SCO4676-4675) (Figure 1A). We therefore investigated the frequency with which these three genes were associated with scr4677. The greatest correlation was observed for SCO4676, which was always located immediately adjacent to scr4677, whenever scr4677 was present (Figure 1A; Table 2); the rest of the intergenic region housing scr4677 did not exhibit the same level of sequence conservation. SCO4676 encodes a putative DNA binding protein with a predicted N-terminal helix-turn-helix motif. Full length homologues were found only in the streptomycetes, but similar predicted DNA binding domains were identified in hypothetical proteins from a wide variety of Firmicutes. In S. coelicolor, SCO4675 was positioned 18 bp downstream of SCO4676. Despite this apparently close coupling, these genes were co-localized in fewer than 10% of Streptomyces chromosomes (Table 2). The function of SCO4675 is unknown, but homologous proteins are encoded throughout the actinobacteria and the fruiting body-forming myxobacteria.

**Table 2:** Conservation of *scr4677* and its surrounding genes§.

	scr4677	SCO4676	SCO4677*	SCO4675*
S. avermitilis	✓	✓	✓	?
S. bingchengensis	✓	✓	?	?
S. coelicoflavus	×	✓	✓	×
S. ghanaensis	✓	✓	?	?
S. globisporus	×	✓	?	×
S. griseoaurantiacus	×	×	✓	?
S. griseoflavus	✓	✓	✓	?
S. griseus	✓	✓	✓	?
S. lividans	✓	✓	✓	✓
S. scabies	✓	✓	×	?
S. sp roseosporus	×	✓	?	×
S. sp. C	✓	✓	?	×
S. sp. e14	×	×	✓	?
S. sp. S4	×	✓	×	?
S. sp. W007	×	✓	?	×
S. sviceus	✓	✓	?	×
S. venezuelae	✓	✓	×	×
S. viridochromogenes	✓	✓	?	×
S. zinciresistens	×	✓	✓	?

§ Conservation is based on similarity scores of >50%, covering >80% of the corresponding protein sequence; conservation for scr4677 required >60% identity over the entire sRNA sequence (conservation in the remaining intergenic sequence was found to be only ∼50%).

* ? reflects the presence of the gene in the chromosome, but not adjacent to scr4677 or SCO4676.

Upstream of *scr4677* is the best characterized gene in the region: *SCO4677*. Like *SCO4676*, *SCO4677* is generally conserved in the streptomycetes, and while it was associated with *SCO4676* and *scr4677* more frequently than *SCO4675,* it co-localized with these genes only ∼30% of the time (**Table 2**). *SCO4677* encodes a predicted anti-sigma factor that, unusually, is not clustered together with a cognate sigma factor- or anti-anti sigma factor-encoding gene. Instead, SCO4677 is predicted to affect the activity of the sporulation-specific sigma factor σ^F^ (encoded by *SCO4035*), and to interact with putative anti-anti sigma factors encoded by *SCO0781* and *SCO0869* [32]. *SCO4677* is a direct target of the developmental regulator BldD [33] and appears also to be controlled by the WD-40 domain-containing protein WdpB (SCO5953) [34]. Expression of *SCO4676*, but not *SCO4675,* is also affected by BldD activity, while the expression of both genes is impacted by the loss of *wdpB* [34].

### scr4677 is Highly Expressed in Diverse *Streptomyces* Species, and Exhibits Glucose-dependent Changes in Expression Profiles

To determine whether *scr4677* sequence conservation in the streptomycetes reflected the existence of an equivalent sRNA gene in these other species, or whether this conservation was simply due to the close proximity of the *scr4677* sequence to *SCO4676,* we took advantage of RNA-seq data that we had generated for three evolutionarily divergent *Streptomyces* species: *S. coelicolor*, *S. avermitilis* and *S. venezuelae* [13]. We found that scr4677 homologues were highly expressed in all three species (**Figure 1B**), strongly supporting a functional role for this RNA molecule. We further validated these findings using northern blotting (**Figure 1C**), and found the sRNA was expressed at different stages of development in each species during submerged or surface growth on MYM medium: in *S. coelicolor*, expression increased throughout development, while in *S. avermitilis,* two transcripts were detected, with highest levels for both seen during the transition from vegetative to aerial growth (48 h); in *S. venezuelae,* high levels of expression were seen throughout the developmental time course, with a smaller transcript also readily detectable during the early stages of vegetative growth (**Figure 1C**).

We had previously confirmed the existence of scr4677 in *S. coelicolor* using northern blotting, with RNA samples isolated from strains grown on either rich (glucose-containing R2YE) medium or minimal medium (MM) supplemented with mannitol [10]. As seen in **Figure 1D**, scr4677 was constitutively expressed during growth on MM with mannitol, but showed more temporal accumulation during growth on rich medium. Smaller transcripts were reproducibly detected in the rich medium-grown samples, but not in those isolated from MM-grown cultures. We reasoned that the smaller-transcript accumulation might be a general response to differences in overall media composition (rich versus minimal media), as they were also observed during growth on MYM (**Figure 1C**). Alternatively, the different transcript profiles may result from differences in carbon source, with the classically rich medium containing glucose (and MYM containing maltose), in contrast to the MM which contained mannitol - a poor carbon source for *S. coelicolor*. To differentiate between these possibilities, scr4677 transcript levels were examined in *S. coelicolor* during growth on soy flour agar medium supplemented with either mannitol or glucose as a carbon source (termed ‘SFM’ or ‘SFG’, respectively). The smaller scr4677 transcripts were observed exclusively in RNA samples isolated from cells grown on SFG (**Figure 1E**), suggesting the appearance of the smaller scr4677 transcripts may be glucose- (or preferred carbon source) dependent. Whether these transcripts are independently expressed sRNAs or stable degradation products has yet to be definitively determined; however, previous investigations have shown that all transcripts appear to share the same 5′ end but differ at their 3′ ends [10], supporting the idea that the smaller transcripts arise as a result of a processing – or premature termination – event.

### Intra-operonic Differences in Expression of *SCO4677*-*4675* in Response to Nutrient Conditions

**Figure 2:**
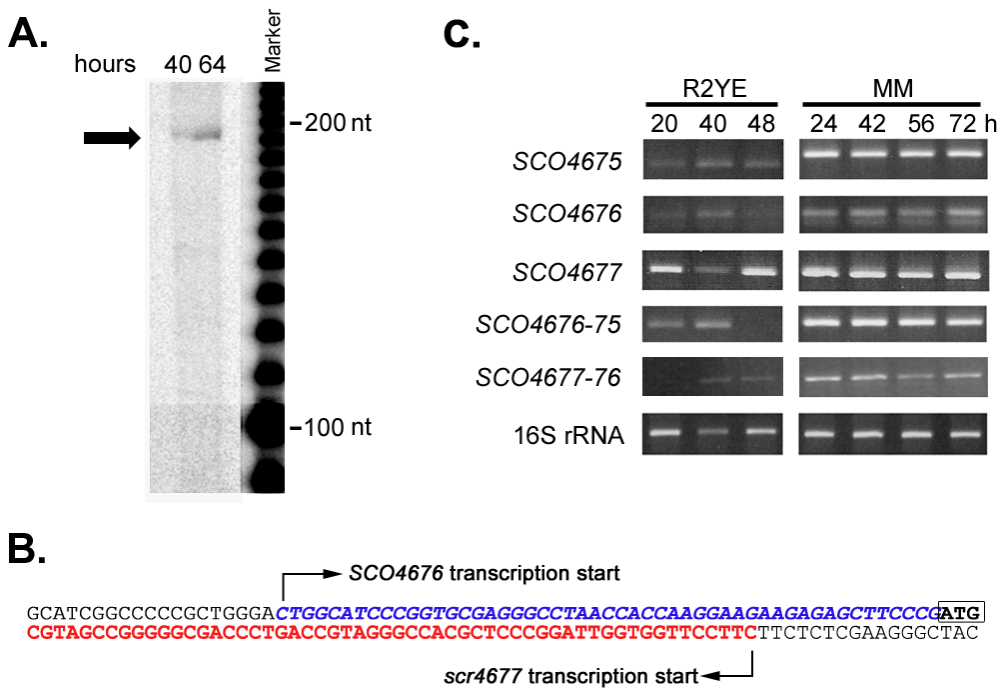
Transcriptional analysis of the genes flanking *scr4677*. A) S1 nuclease mapping of the 5′ end of the *SCO4676* transcript. RNA samples were harvested after 40 or 64 hours of growth on minimal medium supplemented with mannitol. The transcription start site was determined by comparing the size of the protected fragments, indicated with an arrow, to a labeled 10 bp ladder that was run adjacent to these samples. B) Nucleotide sequence upstream of *SCO4676* depicting: the *SCO4676* start codon, bolded and outlined at the extreme 3′ end; the *SCO4676* transcription start site and 5′ UTR indicated in blue italicized text; the *scr4677* transcription start site and sequence illustrated in red text. These two transcripts share 37 nt of sequence overlap. C) Expression profiles for *SCO4677-4675* genes in wild-type *S. coelicolor* following growth on R2YE (glucose-containing rich medium) and MM (minimal medium with mannitol) agar media for the length of time indicated in hours. Transcript levels were assessed using semi-quantitative RT-PCR, with the number of amplification cycles optimized for each transcript (*SCO4675, SCO4676-75, SCO4676, SCO4677*∶28 cycles; *SCO4677-76*∶32 cycles; and 16S rRNA: 15 cycles). 16S rRNA served as a positive control for both the RT-PCR and for overall RNA levels and integrity. These experiments were conducted in triplicate, using at least two independent RNA time-courses.

Given the conservation of the *scr4677* sequence in the intergenic region between *SCO4676* and *SCO4677,* we postulated that scr4677 may regulate the expression of *SCO4676* and/or *SCO4677.* Given that previous 5′ RACE experiments had determined the *scr4677* transcription start site to be a mere 14 nt upstream of the *SCO4676* translation start site [10], we first set out to map the transcription start site for *SCO4676*, to determine whether this mRNA would overlap the scr4677 transcript. Using S1 nuclease mapping with a probe extending ∼150 nt into the *SCO4676* coding sequence, the major *SCO4676* transcription start site was determined to be ∼50 nt upstream of its start codon (**Figure 2A**), consistent with our RNA-seq data, which showed a peak of transcription within this region (**Figure 1B**). This transcript would therefore share 37 nt of sequence overlap with scr4677 (**Figure 2B**). Interestingly, this is roughly equivalent to the size of the smaller scr4677 transcripts, as seen in **Figure 1D**. We also used semi-quantitative RT-PCR to assess the expression of each of *SCO4675, SCO4676* and *SCO4677* during growth on rich medium and MM supplemented with mannitol. As had been seen for *scr4677* (**Figure 1D**), all genes were constitutively expressed over a 72 hour time course on MM-mannitol, whereas they were expressed less highly on rich (R2YE) medium, and showed subtle but reproducible differences in their expression profiles (**Figure 2C**). In particular, *SCO4675* and *SCO4676* were expressed most highly at 40 h, whereas *SCO4677* was expressed more constitutively (relative to the 16S rRNA control).

*In silico* predictions by Castro-Melchor and colleagues [35] had suggested that *SCO4677* and *SCO4676* were co-transcribed. This prediction was borne out by our RNA-seq data (**Figure 1B**), and was further confirmed using semi-quantitative RT-PCR, which showed both *SCO4677*-*SCO4676* and *SCO4676-SCO4675* were co-transcribed (**Figure 2C**).

### scr4677 Impacts Transcript Levels of *SCO4676* and *SCO4677*-*4676*

**Figure 3:**
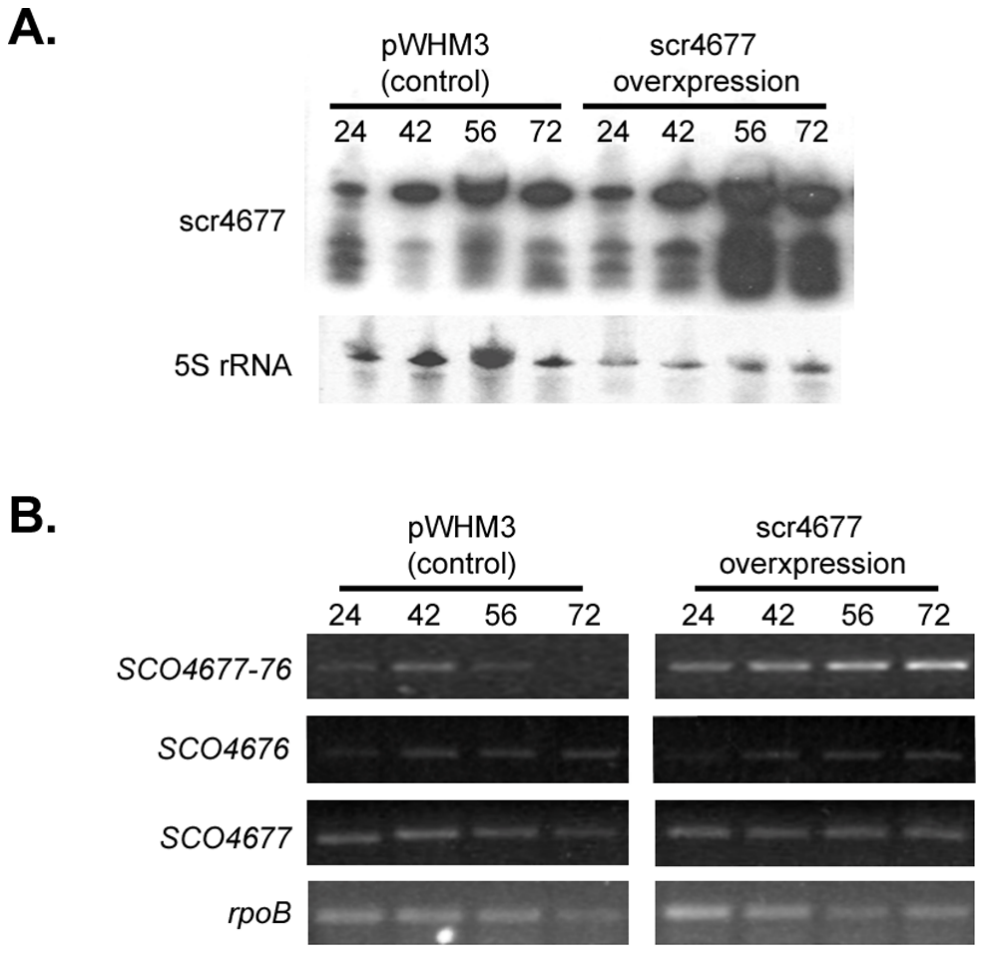
Effect of scr4677 overexpression on *scr4677* flanking genes. A) Northern blot analysis of scr4677 expression when *scr4677* was present on a high copy number plasmid (pWHM3) (right), relative to an empty plasmid-carrying control strain (left), over a 72 hour time course on R2YE (rich) agar medium. 5S rRNA served as a control for RNA integrity and RNA abundance. B) Semi-quantitative RT-PCR was conducted to compare the expression of *SCO4676, SCO4677* and *SCO4677-4676* when scr4677 is expressed from a multi-copy plasmid (right panels), relative to a strain carrying an empty plasmid (left panels). Cycle number was optimized for each reaction (*SCO4676* and *SCO4677*∶28 cycles; *SCO4677-4676*∶32 cycles; *rpoB*: 24 cycles). *rpoB* expression served as a control for RNA integrity and RNA levels. These investigations were conducted using two independent RNA time courses, and at least three technical replicates.

Given that scr4677 would have complete complementary to the *SCO4677-4676* transcript and would share ∼37 nt of sequence overlap with the *SCO4676* transcript, we wanted to determine whether modulating scr4677 expression would impact the abundance of either of these transcripts. We cloned *scr4677*, together with additional upstream and downstream sequence to ensure all regulatory elements were included, into the high-copy number plasmid pWHM3. We introduced this overexpression plasmid into wild type *S. coelicolor* M145 and used northern blot analysis to confirm sRNA overexpression (∼3 fold) during growth on rich R2YE medium, relative to an empty plasmid-containing control strain (**Figure 3A**).

Levels of *SCO4676, SCO4677* and *SCO4677-4676* transcripts were then compared for rich media-grown scr4677 overexpression and control strains. We found *SCO4677-4676* expression levels were reproducibly higher (∼2-3 fold) in the overexpression strain relative to a plasmid-carrying control strain over the entire 72 h-time course (**Figure 3B**), suggesting that scr4677 may stabilize the *SCO4677-4676* polycistronic transcript. Unexpectedly, this did not lead to greater transcript levels for *SCO4676*, which were similar – or even slightly reduced – relative to that of the control strain, suggesting that *SCO4676*-specific transcripts may be less stable when scr4677 levels are increased. We also tested whether the apparent stabilizing effect of scr4677 on the *SCO4677-4676* read-through transcript impacted the overall levels of *SCO4677*; this did not appear to be the case, as these were also similar in both scr4677 overexpression and control strains (**Figure 3B**).

### Effect of RNase III on Gene Expression in the *scr4677* Locus

**Figure 4:**
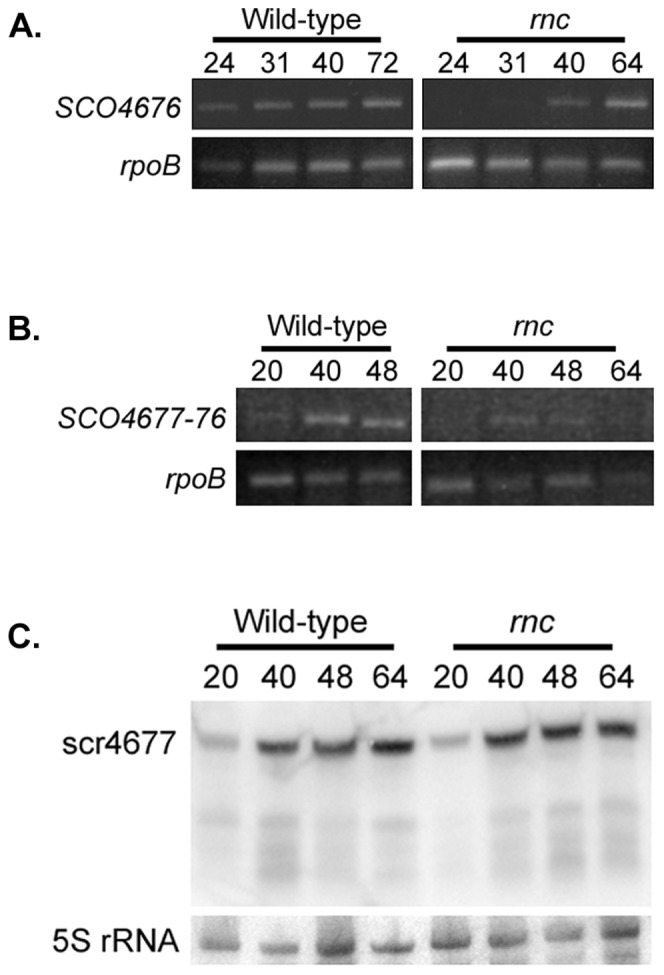
Effect of RNase III mutation on the expression of *scr4677, SCO4676*, and *SCO4677-4676.* (A) Expression of *SCO4676* throughout the developmental cycle of a wild type and isogenic *rnc* (RNase III) mutant strain during growth on rich R2YE medium, as determined by semi-quantitative RT-PCR. *rpoB* served as a positive control for RNA levels/integrity and for the RT-PCR procedure. The number of amplification cycles was optimized for each gene (28 cycles for *SCO4676* and 24 cycles for *rpoB*). B) Same as for A), only examining the level of *SCO4677-4676* read-through expression (using 30 amplification cycles) in wild type and *rnc* mutant strains. C) Northern blot analysis of scr4677 from rich (R2YE) medium-grown wild type and *rnc* mutant strains. In place of *rpoB*, 5S rRNA served as the loading and integrity control.

The location of *scr4677* relative to *SCO4676*, and the extent of complementarity shared by their transcripts, suggested that *SCO4676* transcripts could be targeted for degradation by RNase III upon base-pairing with scr4677. We hypothesized that a strain lacking RNase III (Δ*rnc*) would exhibit increased levels of *SCO4676* mRNA, relative to a wild type strain. To test this, we used semi-quantitative RT-PCR, and found – contrary to expectations - *SCO4676* levels were significantly lower in the *rnc* mutant strain than its parent wild type strain (**Figure 4A**). We also examined the levels of the *SCO4677-4676* read-through transcript, and found that these too were reduced relative to wild type levels (**Figure 4B**). To determine whether these observations could be correlated with changes in scr4677 levels in the *rnc* mutant, we used northern blotting to examine sRNA expression. We found there were no obvious differences in scr4677 transcript levels in Δ*rnc* and wild type strains (**Figure 4C**); the smaller transcripts typically seen during growth on rich medium (see **Figure 1**) were also present at equivalent levels in wild type and mutant strains, suggesting that these transcripts were not generated as a result of RNase III cleavage.

### Investigating the Effect of scr4677 on the Translation of *SCO4676*

Many sRNAs exert their regulatory effects via a translational means. Given the location of scr4677, it was not expected to basepair with the ribosome binding site of *SCO4676*, but it was possible that scr4677 binding to the untranslated region of the *SCO4676* transcript could promote conformational changes in the *SCO4676* mRNA, altering ribosome binding site accessibility and thus *SCO4676* translation. To examine this possibility, we fused a 3×FLAG tag to the C-terminus of SCO4676, and using immunoblotting, attempted to follow its expression in the scr4677-overexpression strain relative to a control-plasmid containing strain. While we were occasionally able to detect a protein of the appropriate size that was never seen in our empty plasmid control strain, this was not consistently observed. We examined expression at different time points, under different growth conditions (*e.g.* minimal medium, SFM, rich R2YE medium), and both with and without precipitation and concentration of cell extracts, but were never able to reproducibly detect the SCO4676-3×FLAG fusion. Therefore we have been unable to draw any conclusions regarding the translational impacts of scr4677 on SCO4676 expression.

### Probing the Biological Role of scr4677 and its Associated Gene Products

**Figure 5:**
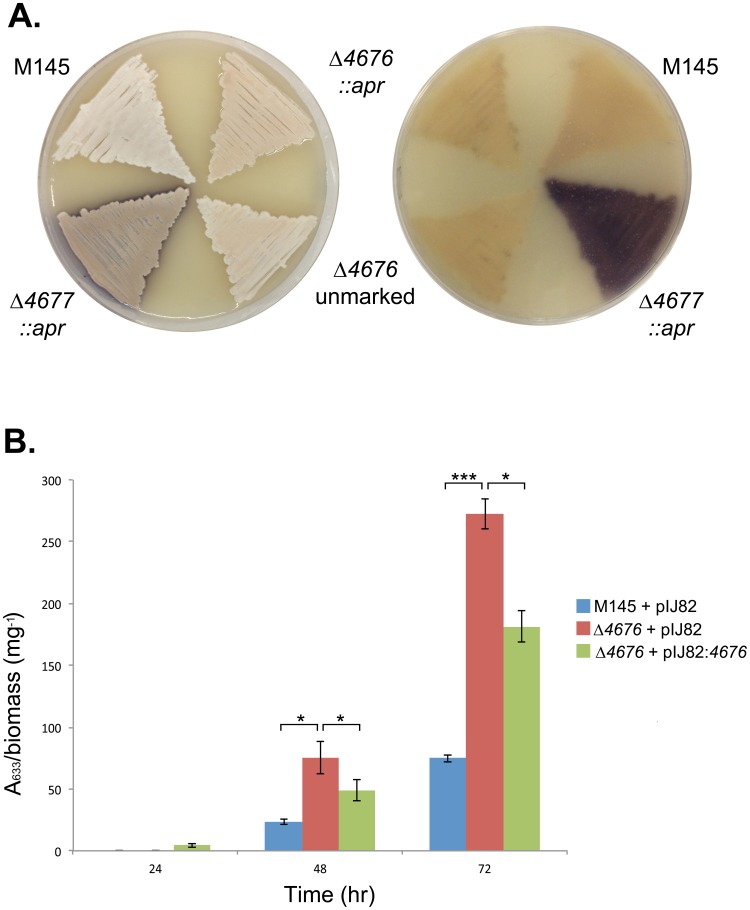
Phenotypic effect of *SCO4676* and *SCO4677* deletion. A) Phenotypic comparison of strains grown on SFM (soy flour supplemented with mannitol) for 48 h. Left image: top of plate; right image: bottom of the same plate. Strain descriptions for the left plate, starting from the top left are as follows: wild type (M145); apramycin resistance-marker containing *SCO4676* deletion mutant (Δ*4676::apr*); Δ*4676* with the apramycin resistance cassette removed to create an in-frame, markerless mutant (Δ*4676* unmarked); and an apramycin resistance-marker containing *SCO4677* deletion mutant (Δ*4677::apr*). B) Enhanced actinorhodin production by the *SCO4676* deletion mutant (carrying the empty integrating plasmid pIJ82), relative to the wild type empty plasmid-carrying strain (M145+pIJ82), as determined by measuring the A_633_, and normalizing to the dry cell weight (in mg). This enhanced antibiotic production could be partially complemented by introduction of wild type *SCO4676* on the integrating plasmid vector pIJ82. The data presented represent the average of three independent cultures, with each analyzed in duplicate; error bars indicate standard error. Asterisks indicate statistically significant differences (* = *P-*value <0.05; *** = *P-*value <0.001).

Given the genetic correlation shared by *scr4677* and its flanking protein coding genes, and the fact that these genes are found in many *Streptomyces* species, we were interested in evaluating the functional role of these gene products. We generated marked and unmarked Δ*SCO4676* mutant strains, with the latter constructed so as to obviate any potential polar effects on the downstream *SCO4675*. We compared the phenotype of Δ*SCO46*7*6* (both marked and unmarked) and wild type strains following growth on a number of different solid media types [rich medium (R2YE+glucose/mannitol), minimal medium+glucose/mannitol, soy flour medium+glucose/mannitol, maltose-yeast extract-malt extract (MYM), supplemented minimal medium solid (SMM with casaminoacids and mannitol) and LB], and found that both mutant strains resembled their wild-type parent during growth on all types of solid media tested (*e.g.* **Figure 5A**). We also tested whether Δ*SCO4676* had any effect on liquid grown-cultures. We consistently observed increased actinorhodin production for the *ΔSCO4676* strain in NMMP liquid medium relative to wild type (the markerless mutant behaved similarly to the *ΔSCO4676* strain; data not shown), and this phenotype was partially complemented by re-introducing wild type *SCO4676* on an integrating plasmid vector (**Figure 5B**).

As scr4677 overexpression led to a slight reduction in levels of *SCO4676*, we were interested in determining whether the phenotype of the scr4677 overexpression strain was similar that of the *SCO4676* mutant, or whether it had additional phenotypic differences that could potentially be attributed to an effect on other target mRNAs. We found the overexpression strain did not exhibit any reproducible phenotypic change relative to an empty plasmid-carrying control strain under any growth conditions tested (data not shown).

As we had established that *SCO4676* was co-transcribed with *SCO4677,* we also generated a deletion strain of *SCO4677*. When grown on SFM agar plates, the *SCO4677* mutant strain overproduced actinorhodin (**Figure 5A**). It also exhibited apparently accelerated sporulation (**Figure 5A**), although some of this darker coloration was due to precocious actinorhodin production. Similar phenotypic effects have been previously reported for a *SCO4677* deletion mutant, and these could be effectively complemented following re-introduction of the wild type gene on an integrating plasmid vector [32].

The altered sporulation profile observed for *ΔSCO4677,* both here and in previous studies [32], suggested that SCO4677 may function in modulating spore development, particularly in light of the fact that SCO4677 interacts with the sporulation sigma factor SigF (which controls the production of the grey spore pigment; [36]). We therefore wondered whether genetic perturbation of either *SCO4676* or *scr4677* might also impact sporulation. We used light microscopy to examine sporulating *ΔSCO4676* and *scr4677* overexpression strains, but could not detect any morphological defects or sporulation abnormalities. We also tested these same strains for altered resistance to heat and a variety of other environmental stresses including cell envelope stress (SDS, EDTA, lysozyme, and vancomycin), oxidative and thiol stresses (hydrogen peroxide and diamide, respectively), and iron starvation (dipyridyl). In all instances, both mutant and overexpression strains behaved similarly to their corresponding wild type control strains.

Thus in summary, our phenotypic analyses revealed that *SCO4677* disruption affected both antibiotic production and sporulation on several media types, *SCO4676* disruption impacted actinorhodin production under specific growth conditions, and modulating *scr4677* levels had no reproducible phenotypic consequences.

### Exploring the Transcriptional Regulation of *scr4677*

**Figure 6:**
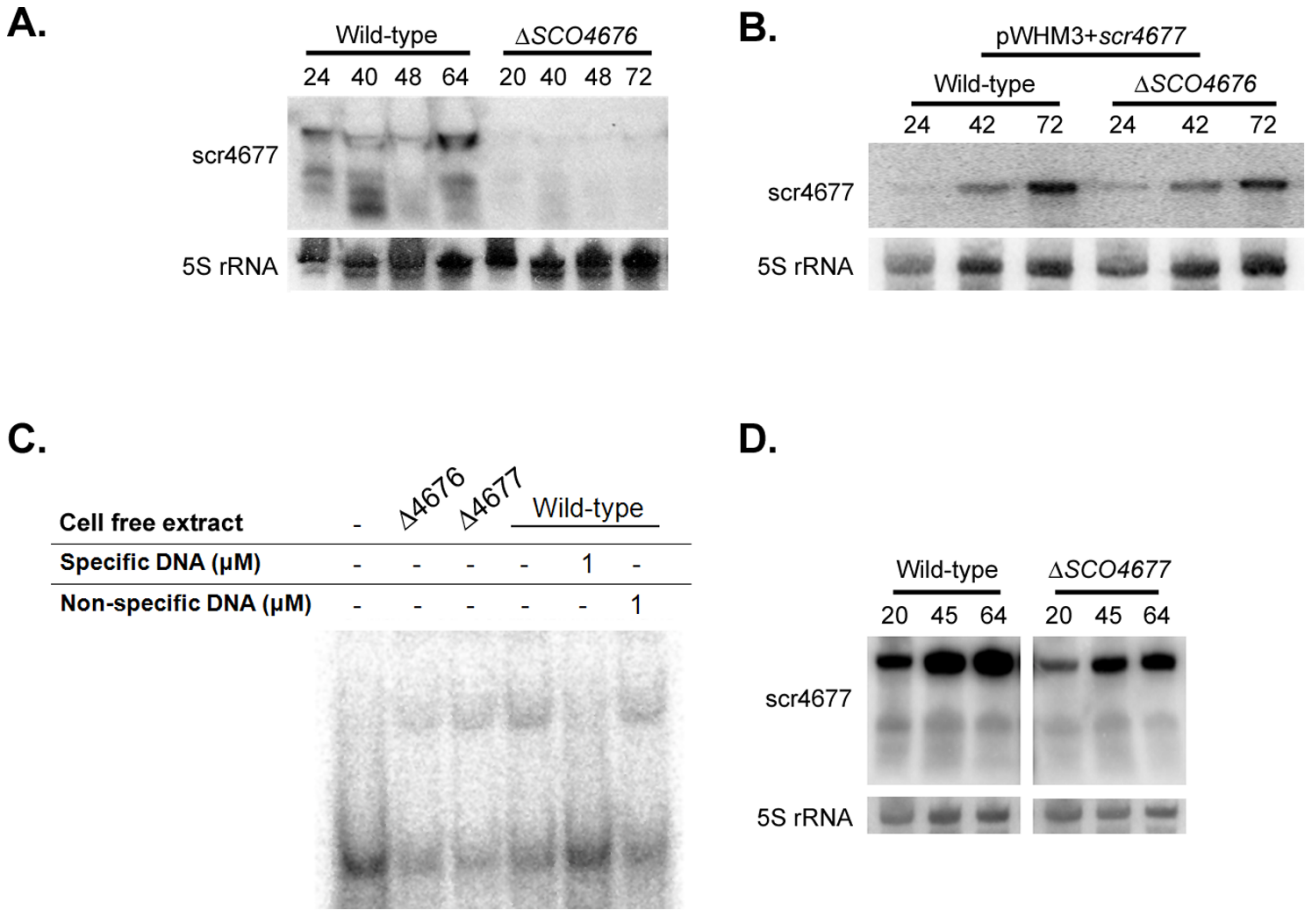
Effect of *SCO4676* and *SCO4677* deletion on the expression of scr4677. A) Northern blot analysis of scr4677 expression in wild type and *SCO4676* deletion (apramycin-containing) strains during development on rich R2YE medium. 5S rRNA was used as a control for RNA abundance and RNA integrity. B) Northern blot analysis of scr4677 as described in A), only wild type and mutant strains carried *scr4677* on a high copy number plasmid (pWHM3). C) Electrophoretic mobility shift assay of a [γ^32^-P] radiolabelled 83 bp probe containing the *scr4677* upstream region (extending from +77 to +159 nt relative to the scr4677 transcription start site), following incubation with cell free extracts from the strains indicated. Binding specificity was tested using excess amounts of specific (non-radiolabeled probe) and non-specific competitor (sequence within *SCO3287*) DNA. D) Northern blot analysis of scr4677 expression during growth on rich (R2YE) medium in a wild type and *SCO4677* deletion mutant strain. As above, 5S rRNA was used as a loading control.

In addition to investigating the regulatory role of scr4677, we also asked whether *scr4677* transcription was controlled by either of its flanking protein-encoding genes (*SCO4676* and *SCO4677)*. Given that SCO4676 is predicted to have a helix-turn-helix motif and may therefore function as a DNA-binding protein, scr4677 expression was first examined in a Δ*SCO4676* mutant strain. The majority of the *SCO4676* coding sequence in this strain had been replaced with an apramycin resistance cassette (in-frame deletion), although the first 21 aa (63 bp) of the N-terminus had been retained to ensure that the *scr4677* promoter region was not affected (**Figure 6A**). Interestingly, we found *scr4677* expression was virtually abolished in the *SCO4676* mutant (**Figure 6B**). This could be due to either the requirement of a functional SCO4676 protein for *scr4677* expression, or the disruption of a regulatory element required for *scr4677* transcription in the *SCO4676* mutant.

To differentiate between these possibilities, we took advantage of our *scr4677* overexpression strain. We tested whether a Δ*SCO4676* mutant strain carrying the scr4677 overexpression plasmid (where *scr4677* had all the necessary elements for effective transcription) was impaired in its expression of scr4677. In this strain, *scr4677* was effectively transcribed (**Figure 6C**), indicating that its expression did not require SCO4676 activity. This instead implied that *scr4677* expression required a regulatory element located ∼80–160 bp upstream of *scr4677* start site, reflecting the sequence that was present in the overexpression construct, but missing in the Δ*SCO4676* mutant. We assessed this possibility by conducting electrophoretic mobility shift assays using a probe spanning this region, together with cell-free extracts from a 48 h plate-grown culture, and determined that there was indeed a protein within these extracts that could specifically interact with this sequence (**Figure 6C**).

We also assessed *scr4677* transcript levels in a Δ*SCO4677* mutant, and found it to be reproducibly expressed at lower levels than in its wild-type parent strain (**Figure 6D**). This suggested that SCO4677 activity might contribute to the full activation of *scr4677* transcription. We tested the ability of extracts harvested from both Δ*SCO4676* and Δ*SCO4677* mutant strains to shift the 83 bp probe described above, and detected shifts equivalent to those observed for wild type extracts (**Figure 6C**). Repeated attempts to isolate the binding protein using affinity chromatography were unsuccessful, due at least in part to low protein concentrations: in *S. coelicolor*, scr4677 was only expressed during growth on solid medium (not during liquid culture), which severely limited our ability to harvest sufficient biomass for such an isolation procedure.

## Discussion

In this study, we sought to begin characterizing the conserved small RNA scr4677. We observed that *scr4677* expression differed depending on nutritional status, in that there were smaller transcripts reproducibly detected in RNA samples isolated from *S. coelicolor* grown on glucose-containing medium. These may represent smaller primary transcripts that are only expressed during growth on optimal carbon sources, although our data suggest they are more likely to be processed products, possibly resulting from generally increased nuclease activity under these growth conditions.

The role of antisense RNAs in modulating the stability of their sense counterparts has been studied most extensively in *E. coli*; however, there have only been a handful of systems examined in which antisense RNAs are expressed within the intergenic sequences of polycistronic mRNAs (*e.g.* [37], [38]). One of the best studied of these is RyhB, an antisense RNA expressed from the intergenic region between *iscR* and *iscS*. During growth under iron-limiting conditions, RyhB stabilizes and promotes the accumulation of *iscR* transcripts (where *iscR* is the first gene of the *iscRSUA* operon), while simultaneously stimulating the degradation of the *iscSUA* polycistronic mRNA [37]. It has been suggested that these dichotomous effects stem from the formation of a stable secondary structural element downstream of *iscR* following RyhB binding [37]: this serves to both stabilize *iscR* transcripts, and promote downstream degradation of *iscSUA* [39]. A converse phenomenon was observed here, where scr4677 enhanced the stability of *SCO4676-77* polycistronic transcripts, and destabilized *SCO4676*-specific transcripts. While our transcription analyses suggested that scr4677 negatively affected *SCO4676* transcript levels, this effect was subtle. This observation is consistent with the emerging view that in bacteria, non-coding RNAs often act to ‘fine-tune’ gene expression (reviewed in [40]).

The differential effects of scr4677 overexpression on the *SCO4676-4677* polycistronic transcript and the *SCO4676*-specific transcript could result from differences in scr4677 binding: the entire 70 nt small RNA sequence has the capacity to base-pair with the polycistronic transcript, while it would share more limited complementarity (37 nt) with the transcript initiating immediately upstream of *SCO4676*. Interestingly, structural predictions (using mFold) for the *SCO4676-4677* intergenic sequence revealed an extended single stranded region ∼70–90 nucleotides upstream from the translation start site of *SCO4676* (**Figure S1**). This region includes an ‘ACAU’ motif, which matches a typical RNase E cleavage site [41]. It is conceivable that scr4677 interaction with this single stranded region alters its conformation, thereby protecting the read-through transcript from RNase E-mediated degradation. The *SCO4676*-specific transcript lacks this region, and association with scr4677 may instead lead to decreased transcript stability.

We initially hypothesized that the destabilizing effect of scr4677 on *SCO4676* could be mediated by the catalytic action of RNase III, since the double stranded RNA that would form as a result of sRNA-mRNA base-pairing would be a reasonable candidate for RNase III-mediated degradation (as reviewed in [42]). We found RNase III had no discernible effect on the levels of scr4677, in line with a recent report showing that in *Bacillus subtilis,* RNase III had little effect on antisense transcript levels [43]. Furthermore, in the *S. coelicolor rnc* mutant, levels of both *SCO4676* and *SCO4677-4676* read-through transcripts were reduced relative to that observed in a wild type strain, suggesting that RNase III was instead required for the expression and/or stability of these transcripts. It has been proposed that RNase III may bind a subset of RNAs and modulate their stability via a non-catalytic mechanism [42]; whether the effect of RNase III on *SCO4676* and *SCO4677-4676* transcript levels is a direct or indirect one remains to be determined.

In considering alternative modes of activity for scr4677, we questioned whether scr4677 could be exerting its regulatory effects on *SCO4676* at a translational level, by altering the structure of the *SCO4676* UTR such that ribosome binding site accessibility was altered. In a situation where ribosome binding to the *SCO4676* mRNA was hindered, this would leave the mRNA transcript susceptible to nucleolytic attack, and could lead to reduced levels of *SCO4676* transcripts. Our data do not exclude this possibility. Such a mechanism has been observed for a number of antisense RNAs (*e.g.* [44]), and as such, we actively pursued testing this possibility for scr4677 and *SCO4676*. We were, however, unable to routinely detect tagged variants of SCO4676 by immunoblotting following during growth for any length of time, on any media tested. We expect the reason for this is simply that the protein is expressed at low levels in the cell, as suggested by our transcriptional analyses (*e.g.* **Figure 1B**).

Both Δ*SCO4676* and Δ*SCO4677* strains produced enhanced levels of actinorhodin relative to their wild-type parents, albeit under different conditions. The *SCO4677* deletion has previously been observed to affect both morphological development and antibiotic production [32]. In this earlier study, SCO4677 was also shown to interact *in vitro* with the sporulation sigma factor SigF, as well as with the putative anti-sigma factor antagonist SCO0869, suggesting that SCO4677 may function as an anti-sigma factor. Mutants lacking *sigF* or *SCO0869* do not, however, exhibit any defects in antibiotic production [32], suggesting that SCO4677 may have additional functions and/or binding partners in the cell.

Collectively, our results suggest the existence of an intricate genetic network linking the scr4677 sRNA, the proposed anti-sigma factor-like protein SCO4677, and SCO4676, a conserved protein that impacts actinorhodin production under specific growth conditions.

## Supporting Information

Figure S1 **Predicted secondary structure of A) the untranslated sequence between** ***SCO4677*** **and** ***SCO4676*** **, and B) the 5′ untranslated sequence of the** ***SCO4676*** **-specific transcript.** In A) the red text corresponds to the *SCO4676* untranslated sequence (shown in B), while the blue text indicates an extended single stranded region. The four nucleotides highlighted with the green box indicate a typical RNase E cleavage site [(A/G)NAU].
(TIF)

Table S1 **Oligonucleotides used in this study.**
(XLSX)
