# Supplementary figures and images for: Complex Intra-Operonic Dynamics Mediated by a Small RNA in Streptomyces coelicolor
Source: PLoS One. 2014 Jan 20;9(1):e85856. doi: 10.1371/journal.pone.0085856 (PMC3896431; doi:10.1371/journal.pone.0085856)

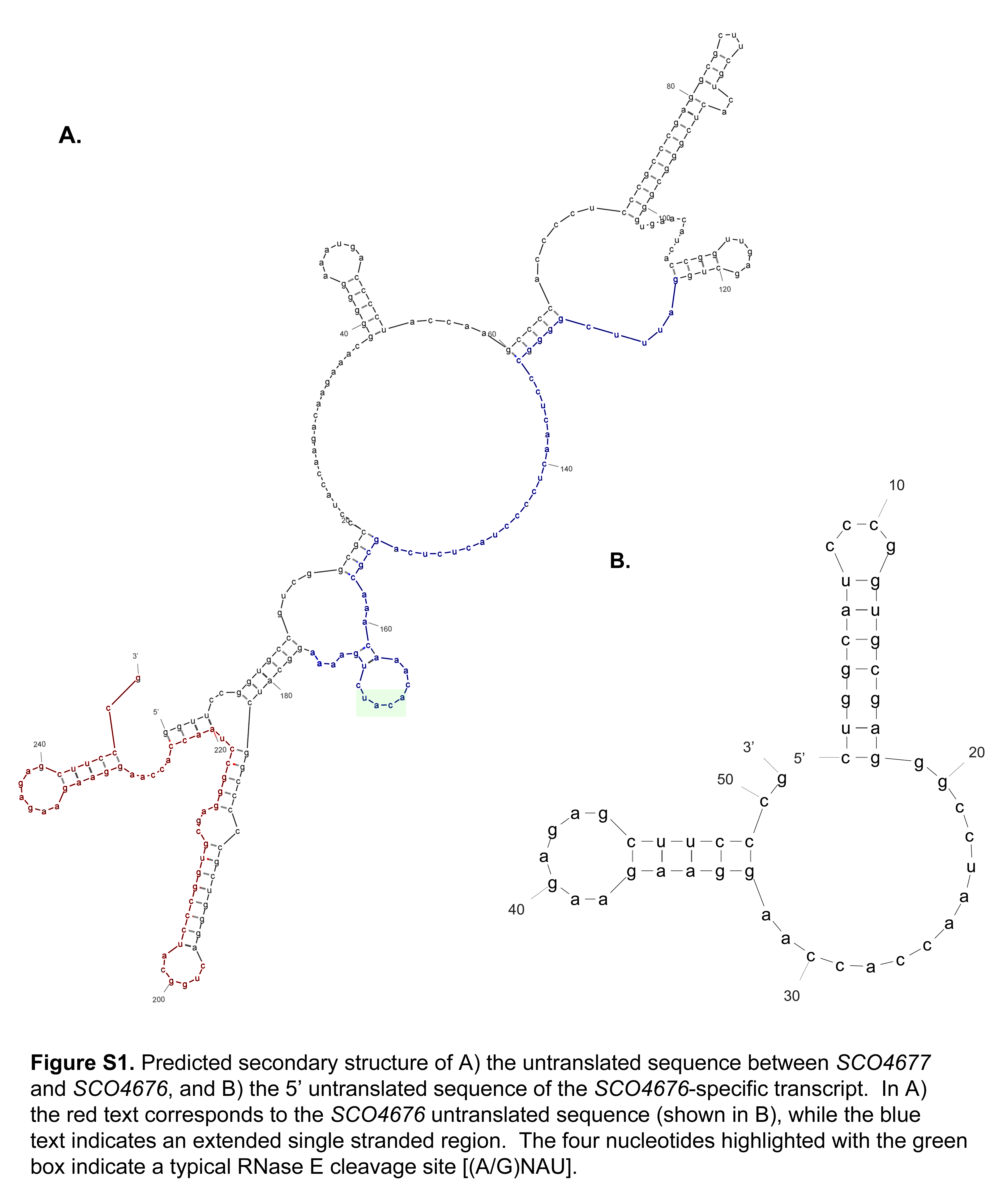

Supplement: Figure S1 — Predicted secondary structure of A) the untranslated sequence between SCO4677 and SCO4676 , and B) the 5′ untranslated sequence of the SCO4676 -specific transcript. In A) the red text corresponds to the SCO4676 untranslated sequence (shown in B), while the blue text indicates an extended single stranded region. The four nucleotides highlighted with the green box indicate a typical RNase E cleavage site [(A/G)NAU]. (TIF) [file pone.0085856.s001.tif]
